# Human-animal relationships in older persons: from pet companionship to animal-assisted interventions. A position statement

**DOI:** 10.1007/s40520-026-03386-4

**Published:** 2026-04-11

**Authors:** Andrea Ungar, I. Ambrosino, G. Rivasi, L. Ceolin, G. Bellelli, E. Bisconti, M. C. Catalani, A. Cherubini, L. Colombo, L. Contalbrigo, A. M. Cotroneo, C. Mariti, L. Mechelli, M. Melosi, A. Morandi, F. Mutinelli, C. Mussi

**Affiliations:** 1 Division of Geriatric and high-intensity Care Medicine and Geriatric Cardiology, University of Florence, Azienda Ospedaliero-Universitaria Careggi, Florence, Italy; 2 Associazione Italiana Geriatri e Veterinari (VETeris), Florence, Italy; 3 School of Medicine and Surgery, Acute Geriatric Units, University of Milano-Bicocca, IRCCS San Gerardo dei Tintori, Monza, Italy; 4 Dog handler, Arezzo, Italy; 5 Past President of Italian Society for Animal Behavioural Sciences, Cremona, Italy; 6 Geriatria, Accettazione geriatrica e Centro di ricerca per l’invecchiamento, IRCCS INRCA, Ancona, Italy; 7 Department of Clinical and Molecular Sciences, Università Politecnica delle marche, Ancona, Italy; 8 MSD Animal Health, Milan, Italy; 9 National Reference Centre for Animal Assisted Interventions, Istituto Zooprofilattico Sperimentale delle Venezie, Padova, Italy; 10 Division of Geriatric Medicine, Azienda Sanitaria Locale “Città di Torino”, Torino, Italy; 11 Department of Veterinary Sciences, University of Pisa, Pisa, Italy; 12 Department of Veterinary Medicine, University of Perugia, Perugia, Italy; 13 President of National Association of Italian Veterinary Doctors, Cremona, Italy; 14 Department of Clinical and Experimental Science, University of Brescia, Brescia, Italy; 15 Azienda Speciale Cremona Solidale, Cremona, Italy; 16 Department of Biomedical, Metabolic and Neuroscience, University of Modena and Reggio Emilia and Baggiovara Civil Hospital, Modena, Italy; 17 Florence, Italy

**Keywords:** Human-animal relationship, Older adults, Pet companionship, Animal-Assisted Interventions, Dog-assisted interventions, Quality of life, Dementia, Chronic care

## Abstract

The rising awareness of how animal companionship enhances human well-being has drawn significant attention from people of all ages. Literature data about the impact of human-animal relationships is still limited by the number of studies and the settings explored, especially among older adults. However, encouraging results were provided by some preliminary studies investigating pet ownership and AAI in old age. In this manuscript we provide a detailed overview of the effects of the human-animal relationship in older people. We review data on the benefits of pet ownership in later life and on Animal-Assisted Interventions (AAI) as a non-pharmacological strategy to support traditional approaches to the most prevalent disabling conditions in the older population, focusing on the technical requirements that guarantee the quality and safety of AAI. Indeed, AAI may allow for the advantages of the positive effects of the human-animal bond in the older population without the challenges of pet companionship ownership, which may not always be suitable for some older adults.

## Introduction

The World Health Organization (WHO) defines health as “a state of complete physical, mental and social well-being, and not simply the absence of disease”. Therefore, health promotion can be defined as the process of empowering people to increase control over their health and to improve their self-efficacy to develop healthy habits [1].

Health is a positive concept that enhances personal and social resources, as well as physical capabilities. This is where the Bio-Psycho-Social model originates: there is no health if all these three areas are not well balanced with each other [2].

According to research by Rudnicka et al. (2020) on healthy ageing, evidence supports that regular physical activity, a nutrient-dense diet, and avoidance of tobacco and alcohol are key strategies for maintaining health [3]. In addition to the above, in the last years, pets have emerged as an additional factor that might positively impact individuals’ health and well-being [4]. Available evidence investigating the beneficial effects of living with pets mainly consists of retrospective observational studies [5].However, experimental designs typically employed for traditional diagnostic and therapeutic interventions may not be feasible in this field. Consequently, establishing a causal link between cohabiting with animals and improvements in physical, psychological, and mental health remains challenging. Nonetheless, evidence of good quality and narrative research exist, providing support to the beneficial effects of living with animals [6].

These data will be illustrated and discussed in the present position statement, aimed to provide an overview of the effects of the human-animal relationship in older people. The authors examine the benefits of pet companionship for older adults and the role of Animal-Assisted Interventions (AAI) as a non-pharmacological strategy. It highlights how AAI supports traditional care for age-related disabilities, emphasizing the technical standards necessary to guarantee safety and quality. Indeed, AAI may allow for the advantages of the positive effects of the human-relationship in the older population without the challenges of pet ownership, which may not always be suitable for some older adults.

## Pet companionship benefits in older people

**Fig. 1:**
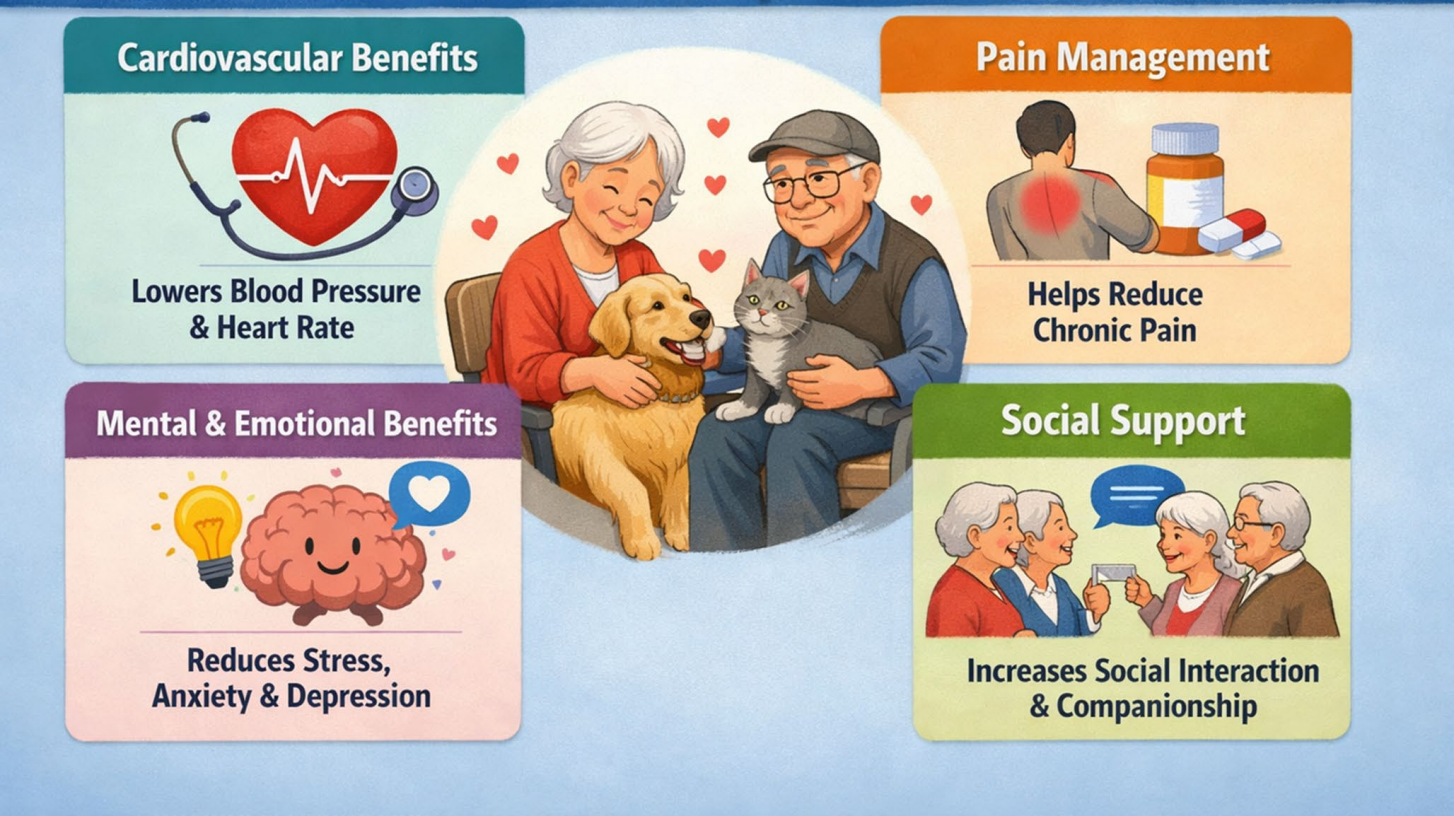
Benefits of animal-assisted intervention in the older population (Figure created with Chat-Gpt)

As the most common form of human–animal interaction, pet comanionship can significantly improve the well-being of older people, even in the presence of prevalent comorbidities like cardiovascular disease, chronic pain, and depression (Fig. 1) [7].

### Cardiovascular benefits

Cardiovascular diseases (CVD) represent the leading cause of morbidity and mortality, particularly above the age of 65 [8]. Some of the strongest research evidence on the impact of pet companionship on human health and well-being comes from data about cardiovascular risk. Scientific evidence highlights that sedentary behavior, hypertension, obesity or overweight, coronary heart disease, along with abnormal lipid profiles increase cardiovascular risk [9].

Living with pets may influence cardiovascular risk factors, potentially reducing individual cardiovascular risk [10]. Dogs may encourage walking and the adoption of a more active daily routine [11], favouring adherence to weight loss programs in adults [12]. Living with animals seems to reduce systolic and diastolic values, pulse and mean blood pressure in both hypertensive and normotensive subjects [13]. The physiological mechanisms involved might be related to vagal tone increase and reduction of sympathetic activity [14]. Furthermore, some studies support the hypothesis that pet-ownership may reduce stroke and cardiovascular mortality [13, 15], and may positively impact cholesterol and triglyceride levels [16], although a more detailed analysis of possible confounders is needed to confirm these associations [17, 18].

### Pain perception and control benefits

**Fig. 2:**
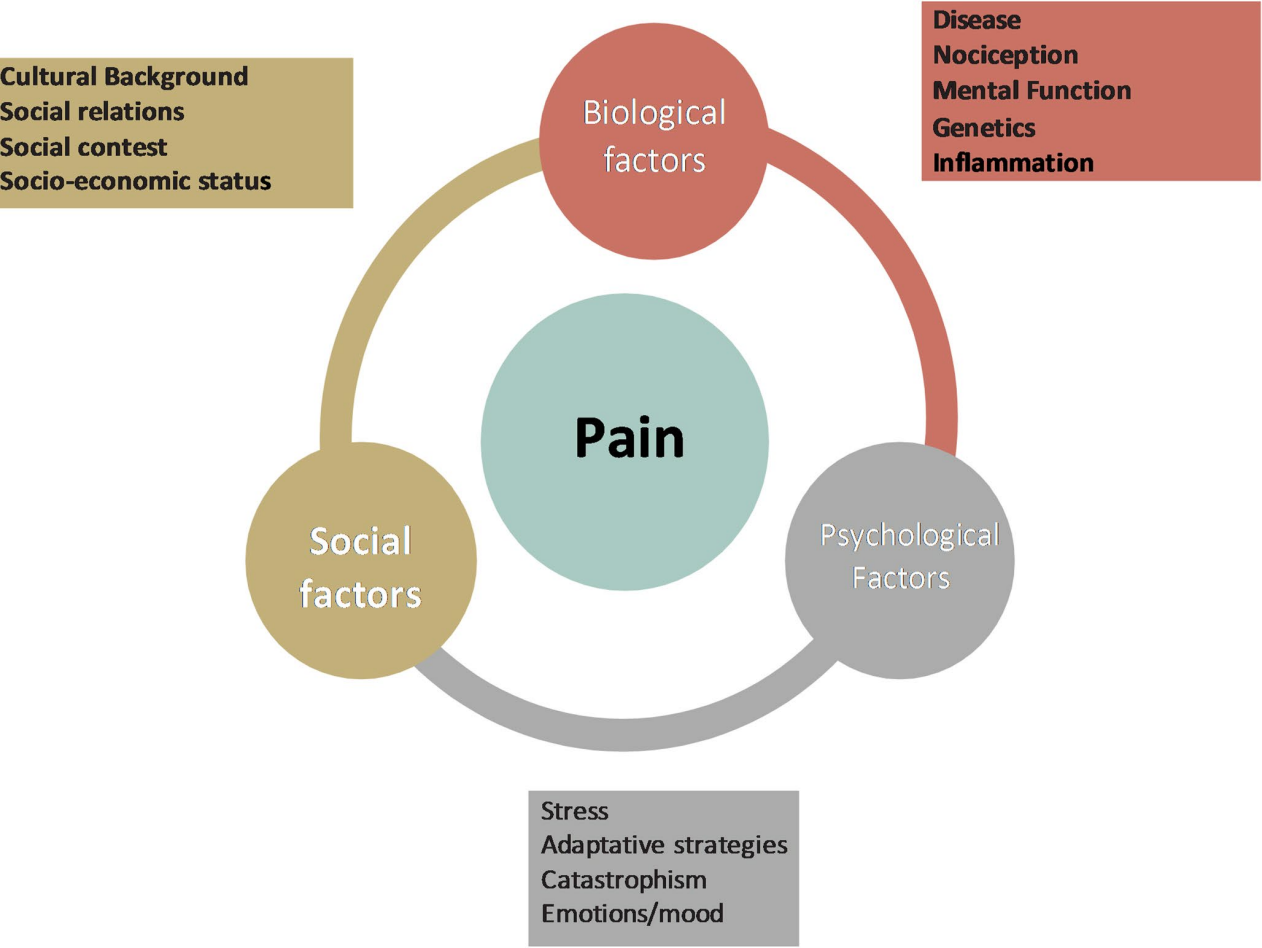
Factors influencing pain perception

Pain is a subjective experience influenced by biological (inflammation, genetic factors, nociceptive functions, concomitant diseases etc.); social (social context and network, economic status) and psychological variables (emotions, mood, stress level and individual resilience) (Fig. 2) [19]. Chronic pain has relevant prevalence at old age and often deteriorates quality of life of older adults [20].

Living with pets seems to have a positive impact on factors that influence pain perception [21]. Indeed, pets favour the adoption of a more active lifestyle [22, 23] that may help to preserve self-efficacy and functional autonomy, reducing inactivity-related chronic pain perception [21]. The relationship with a companion animal may help to relieve stress, improve sleep routine and shift the owner’s attention from pain to the animal [24]. Moreover, companion animals act as social facilitators contrasting loneliness and social activity restrictions, which are common in older people affected by chronic pain [25]. Finally, pets are often described as a source of comfort, support and protection with beneficial effects on individual’s mood [21]. Chronic pain and depressive symptoms interact in a relationship of mutual amplification, where affective disorders lower the pain tolerance threshold and persistent pain fuels social isolation and psychological decline [26]. The support provided by the human-animal relationship can act as an effective non-pharmacological mediator, improving emotional resilience and reducing the subjective perception of pain [27].

### Mental and emotional benefits

Ageing is one of the major risk factors for cognitive decline [28]. Therefore, non-pharmacological strategies promoting well-being, via the improvement of cognitive and functional performanceare gaining increasing attention [29, 30].

*Memory and cognitive performance*. Some data indicate that pet-ownership in adult age might improve cognitive performance in later life. Older people living with animals show better cognitive function, particularly as regards verbal learning and memory. Furthermore, there is evidence demonstrating a reduction of behavioural symptoms in older patients with dementia [31].

*Depression*. Living with animals can prevent or reduce depressive symptoms through multiple mechanisms: [29, 31].

1. Company and support: loneliness is one of the major risk factors for depression. Living with animals may provide companionship and emotional support for people with limited social interactions.
2. Daily routine: Caring for an animal requires well-defined daily schedules that may stimulate cognitive function and reduce inactivity and apathy associated with depression [31].
3. Social role: one of the most frequent triggers of depression at old age is the loss of social role within the family and the society. Caring for an animal may potentially restore this role, as pet-owners feel themselves useful and needed for their companion animal [29].

### Social support

Loneliness is a major risk factor for disabling conditions like depression and anxiety. The COVID-19 pandemic has exacerbated feelings of loneliness, abandonment and insecurity in many older individuals. Available studies investigating the benefits of living with a pet during the lockdown periods are mainly qualitative (e.g., surveys and narrative-based medicine) [32, 33], however their results suggest that companion animals have provided emotional support in the stressful situation of the pandemic [34]. According to a survey investigating pets’ impact on everyday life of older adults during the COVID-19 pandemic, many participants “have no idea how [they] would cope with the stress [if they] were without pets” [35].

## Recommendations for pet-ownership at old age

Pets can offer many benefits to older people, but caring for a pet comes with some responsibilities and challenges because all their physiological and ethological needs should be satisfied to guarantee their well-being. Therefore, some recommendations are mandatory for a successful adoption process.

1. Assessment of the context: key factors to consider include the prospective owner’s, health status, and economic situation, as well as the home environment and family context in which the animal will live. It is also important to evaluate the presence of additional caregivers who could assist with pet care if needed.
2. Selection of the most appropriate species: this decision should take into account the individual’s previous experience with pets, personal preferences and the ability to align the physiological and ethological needs of the species with the older person’s physical and cognitive abilities, as well as their lifestyle. “While dogs and cats remain the most common choices for older adults, other animals—such as birds or small rodents—may be more suitable for those with limited financial resources or a desire for minimal lifestyle adjustments [36, 37] .
3. Matching the pet with the older person: the individual characteristics of the pet – such as age, breed, health status, level of socialization, education, training level and any other special needs - should be carefully assessed in relation to both the environmental context and the specific traits of the prospective owner. This helps ensure an optimal match between pet and owner.

### Cat Adoption by older persons: considerations and suggestions

The human-cat bond has been found to alleviate symptoms of anxiety, depression and fear. However, persons who lead an active lifestyle, spend a lot of time away from home, or travel frequently may not be ideal candidates for cat ownership. Given the average feline lifespan of approximately 15 years, the adopter’s age, health status and the availability of long-term family support for pet caretaking should be considered, especially when deciding between adopting a kitten or an adult cat [38].

Adult cats usually exhibit calmer and more predictable behaviour, which is more compatible with the routine of older persons [39]. By contrast, kittens and young cats often display high levels of activity, including scratching and chasing behaviours, which may pose challenges or even fall risks for older owners [40].

The home environment plays a central role in ensuring the cat’s psychophysical well-being [41]. Cats require sufficient space to move, explore and access separate areas for water, food and litter.

The litter box should be easily accessible and daily cleaning is recommended. Resting areas should be available in different rooms and at different heights [42].

If outdoor access is allowed, appropriate safety measures must be implemented to prevent the cat from reaching streets or other hazardous areas.

### Dog Adoption by older persons: considerations and suggestions

Dogs can promote physical activity, social interactions, and outdoor engagement, while also offering emotional support to their owners. However, older adults with balance and gait impairments, musculoskeletal or mental disorders or reduced autonomy might not be suitable for dog ownership, due to a possible increase in falls.

Given the diversity among dog breeds, personality traits and care requirements can vary significantly. Therefore, it might be necessary to ensure the presence of additional pet caregivers who can assist the older person with handling and general care. Well-socialized, trained adult dogs are generally the most appropriate choice for older individuals [43]. In these cases, collaboration with a veterinarian experienced in dog behaviour is highly recommended to guide the matching process. This professional can provide tailored advice to help the new owner develop a positive relationship with the dog and to adapt their daily routine to include playtime, physical activity, and social interaction. In contrast, puppies demand significant time and energy for training, exercise and socialization, which may exceed the capabilities of some older adults. In addition, when considering pet adoption, it is crucial to take into account the animal’s expected lifespan as well as the possibility of future changes in the owner’s living situation, to ensure that the animal will continue to receive appropriate care in case the older person becomes unable to do so.

## Benefits of Animal-Assisted Interventions in older adults

“Animal-Assisted Interventions” (AAI) is an umbrella term encompassing services delivered by professionals, involving domestic animals for therapeutic, educational or recreational purposes. These interventions are categorized based on their specific aims and objectives. Animal-Assisted Therapy (AAT) is a personalized intervention designed to complement traditional therapeutic strategies. Animal-Assisted Education (AAE) is designed to support individuals’ skills development in areas such as cognition, social functioning, and personal growth in the living environment; Animal- Assisted Activities (AAA) are intended to enhance the individuals’ quality of life through recreational programs benefiting both the human participants and the animals involved. AAI necessitate careful planning and the collaboration of professionals tailored to the specific goals of the intervention and the characteristics of the individuals involved [44].

Older persons often face the challenges of multimorbidity and polypharmacy, which can lead to disability and increased risk of institutionalization, thus negatively impacting their quality of life 45. AAI are based on affective-emotional motivation and psychological stimulation and represent a promising approach for older adults, addressing various aspects of their well-being and providing benefits across multiple domains of care, as described in the following paragraphs.

### Animal-Assisted Interventions in older persons living with dementia

To date, most studies investigating the effects of AAI at old age have focused on older adults with dementia [46]. Available evidence suggests that AAI can reduce behavioural and psychological symptoms, even in the later stages of the disease. Specifically, agitation, hyperactivity, aggressive behaviours and irritability appear to be reduced in both frequency and severity through AAI, suggesting their potential role as complementary treatment [47–56]. Some studies also indicate that AAI may stimulate cognitive functions, improving attention, motivation and psychomotor skills including balance [55]. However, available research does not report substantial benefits on cognitive performance and autonomy in daily activities [49, 54, 56].

Individuals with dementia frequently experience anxiety and depression [57]. Some studies have highlighted that positive emotions elicited through the interaction with animals may significantly reduce depressive symptoms and anxiety [46, 53–56]. Animals are often described as social lubricants, fostering social interactions and communication [46, 48, 50, 51, 58–60].

**Fig. 3:**
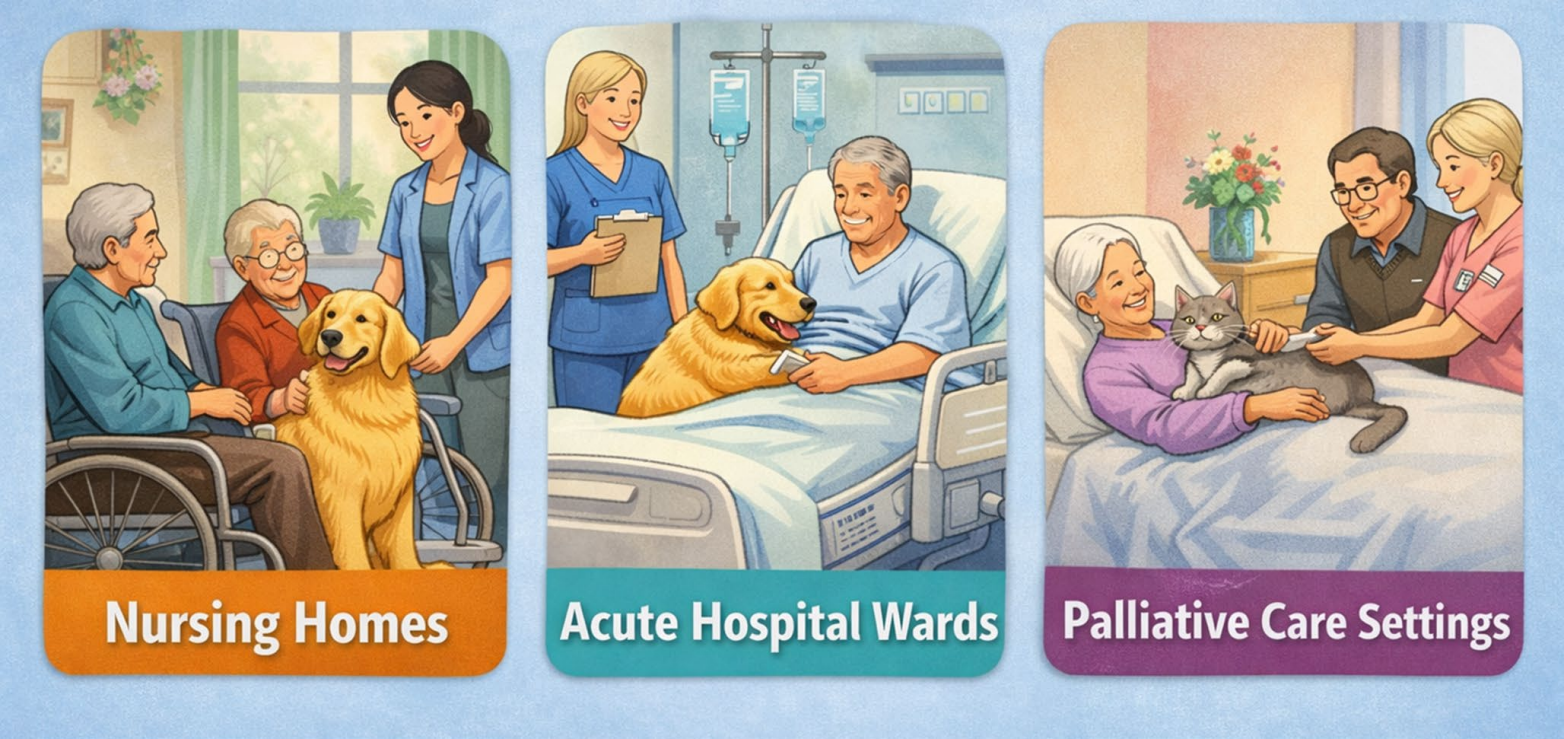
Settings for the application of animal-assisted interventions in older persons (created with Chat-GPT)

Moreover, benefits of AAI may extend to caregivers, including family members and healthcare professionals, by alleviating stress and reducing the burden of care [61]. (Table 1; Fig. 3)

**Table 1:** Applications of Animal-Assisted Interventions in older persons

Setting	Population	Possible benefits
Nursing Homes	Older persons with dementia	• Reduction of behavioural symptoms • Reduction of anxiety and depressive symptoms • Increased physical activity • Balance improvement • Increase of social interactions
	Institutionalized cognitively unimpaired individuals	• Reduction of loneliness • Reduction of depressive symptoms
	Older persons with mental illness	Improvement of social functioning
Palliative Care	Patients affected by end-stage diseases (dementia and other diseases)	• Reduction of anxiety • Reduction of loneliness • Pain relief
Hospital	Hospitalized older patients	• Improvement in anxiety and mood • Reduction of hospital-related stress

### Animal-Assisted Interventions in the nursing home setting

Some studies involving institutionalized cognitively unimpaired older adults have described a positive impact of AAI on different components of individual’s well-being [62–63]. Beneficial effects have been observed in relation to depressive symptoms [60, 64–66], loneliness and quality of life, especially among subjects with a history of pet-ownership or a strong emotional bond with animals [67, 68]. The effects of AAI in institutionalised older individuals with mental illness remain less clear. Indeed, some studies indicate improvements in social functioning and communication abilities [69, 70], while the potential benefits on behavioural symptoms and mood remain doubtful [46].

### Animal-assisted interventions in hospitalized older adults

Hospital stays can be very stressful for older individuals [71]. Existing literature on institutionalized older individuals suggest that AAI programs might have favourable effects on hospital-related agitation and anxiety. However, at present, only a few small pilot studies have evaluated the impact of AAI in older hospitalized individuals. The results indicate a substantial reduction of in-hospital stress and anxiety, with possible beneficial effects also on mood and pain perception [72–77].

### Animal-assisted interventions in palliative care

Preliminary data suggest that AAI may offer a valuable non-pharmacological strategy supporting palliative care for older persons. Indeed, the human-animal relationship can help reduce feelings of loneliness, shift attention from suffering to the animal and generate positive emotions, thereby alleviating anxiety and pain, also in older persons with advanced dementia [78, 79]. Again, these beneficial effects appear to be more pronounced in individuals with a history of pet ownership [80].

### Limitations of current evidence and future perspectives

Despite promising preliminary results, available evidence on AAI in older persons remains scarce. Moreover, some relevant limitations should be acknowledged, including the following [46, 47, 81]:

- Small sample sizes of the study populations;
- Lack of standardization in interventions, regarding the type, duration and frequency of AAI programs;
- Heterogeneity in study outcomes and instruments used to evaluate the effects of interventions;
- Study design limitations, including the lack of “active” control groups receiving alternative interventions, that could help clarify the specificity of AAI effects and the possible confounding influence of receiving increased attention from therapists;
- Short follow-up periods and the lack of mid/long-term reassessment to investigate the persistence of the effects;
- Insufficient data on potential confounders and other variables that may guide the selection of appropriate candidates for AAI programs;
- Lack of research on AAI in home-based settings.

Preliminary research suggests that AAI could offer additional benefits for older persons, that should be addressed in future studies. In individuals with motor impairments and/or neurological diseases (e.g., previous stroke, Parkinson’s disease), AAI may play a complementary role to physiotherapy and neurological rehabilitation [82], encouraging patients’ participation in task-specific motor activities and helping to preserve functional autonomy. Specifically, in patients with Parkinson’s disease, AAI may promote physical activity and social interactions, potentially improving physical performance and psychological well-being [47]. Finally, AAI could serve as an adjunctive therapy for individuals suffering from chronic pain conditions [83, 84].

## Regulation and organizational issues of Animal-Assisted Interventions for older persons

In Italy, AAI were officially regulated in 2015 through the establishment of National Guidelines aimed to standardize the service [44].

### The Multidisciplinary team for animal-assisted interventions

The implementation of AAI requires a multidisciplinary team, whose coordinated work is essential for the planning and delivery of the intervention. The composition of the team should be tailored to the characteristics and purposes of the intervention, as well as to the needs of the older persons involved [44]. According to the Italian National Guidelines for AAI, team members should be qualified, have specific training in AAI and be officially certified and registered in national database of AAI professionals, known as “Digital Pet” (www.digitalpet.it). In Animal Assisted Therapy (AAT) and Education (AAE) projects, the multidisciplinary team should include the following roles: project leader, veterinarian with AAI expertise, animal handler (Table 2).

**Table 2:** Multidisciplinary team for animal assisted intervention (AAI)

Multidisciplinary team for AAI	
Project leader	Responsible for the coordination of the team, definition of project objectives and evaluation of outcomes;
Veterinarian with AAI expertise	Selects the animal-handler team, in collaboration with the project leader, ensure compliance with health and behavioural standards for the animal and develop management protocols throughout the intervention. The veterinarian is also responsible for the regular monitoring of health status, behaviour and well-being of the animal using dedicated clinical records [31].
Animal handler	Responsible for the daily care and management of the animal, mediates interactions between the animal and the participant and ensures the animal’s welfare throughout the intervention [31].

On-site, a diamond approach is mandatory, meaning that the healthcare or educational professional involved with the participant and the animal handler should work in close collaboration.

In Animal Assisted Activities (AAA), an activity leader is responsible for the planning and coordination of the project. Finally, the animal is recognized as a member of the multidisciplinary team. According to Italian National Guidelines, dogs, cats, rabbits, donkeys and horses can be involved in AAT and AAE, while any domestic species can be involved in AAA projects.

### AAI and the therapeutic, educational or recreational relationship with animals in older persons

AAI are centred on the human-animal relationship, consisting of structured series of positive interactions between the animal, the participant/patient and members of the AAI team [85]. Unlike pet ownership, which is characterized by spontaneous and uncontrolled interactions shaped by personal habits and individual’s behaviours, AAI are guided by professionals to align with specific therapeutic, educational, or recreational goals. It is, therefore, essential to distinguish the therapeutic value of the human-animal relationship within the context of AAI from the general benefits associated with pet ownership. Indeed, owning a pet may offer emotional support and companionship, but the quality and outcomes of that relationship are highly variable and not always beneficial. In fact, in cases of poor compatibility between the owner and the pet, the relationship may develop a negative valence, potentially leading to stress [86]. AAI programs for older persons mainly focus on relational and psychological dimensions such as emotional care, affective engagement, cognitive leisure and social functioning and interactions [87]. Referential and observational activities that do not include the active involvement of participants should be limited, as they may fail to engage individuals with limited attention and motivation, sensory impairments or mood disorders [46]. Moreover, programs that incorporate complex or physically demanding exercises may be inappropriate as they can induce feelings of inadequacy and frustration in older participants [88, 89].

### The animal/handler team in AAI programs for older persons

Animals involved in AAI programs for older persons should undergo specific training as older persons often present with comorbidities and/or severe frailty. The suitability of animals should be carefully evaluated to ensure the absence of behavioural problems, taking into consideration temperament, level of socialization and obedience skills [90]. Education and training should be aimed to enhance social skills and maintain motivation and engagement of the animal. Regular training sessions led by qualified professionals should be performed and should employ positive reinforcement techniques, explicitly avoiding any coercive methods, in line with the European Convention for the protection of companion animals [91]. Among species participating in AAI for older adults, dogs are the most commonly involved [92]. These animals should meet specific behavioural requirements to ensure safety and effectiveness throughout the intervention.

### Behavioural characteristics of dogs involved in dog-assisted interventions (DAI) for older persons

Dogs involved in DAI for older persons should express pro-sociality, docility, tolerance to close contact and handling, adaptability, reliability and predictability, combined with high response thresholds. All these behavioural traits are influenced by genetic and early experiences in an environment rich of stimuli [93]. During DAI sessions, relational skills of dogs have a pivotal role. This allows to build a collaborative relationship with no need of repeated positive reinforcements, e.g. food rewards, and maintaining the dog in a state of moderate arousal during the entire session [94].

### Dog Education and Training: recommendations

It is advisable that dogs selected for being involved in DAI have an attitudinal predisposition, that education and training programs should strengthen through appropriate, coercion-free methods, based on learning theory and on the possibility to create a good relationship with the human partner and to establish positive interaction with human beings. Moreover, the training phase guarantees the development of a proper partnership with the handler, i.e. the dog ability to work with the handler and to perform specific tasks [95]. Role-playing and simulation of scenarios during training can be useful additional tools to facilitate the learning process.

The training program for dogs involved in DAI should include some key elements that consider the hazards for frailer older persons in the interaction with the dog. The dog should learn to perform only some behavioural patterns avoiding others: i.e. hand shaking should be avoided, with preference given to nose touch to hand and other verbal commands such as laying down and staying, staying in a sitting position, moving from one place to another. Some behavioural scripts are useful to guarantee appropriate behaviour in the social context of the intervention. Furthermore, synchrony between the dog and the handler should be developed through the perception and integration of multimodal communicative signals [96].

### Dog welfare monitoring

A successful AAI program always includes animal health and welfare monitoring strategies to protect the animal well-being. According to the Italian National Guidelines, the veterinarian with expertise in AAI establishes the welfare monitoring plan based on behavioural and physiological indicators considering also environmental parameters. The monitoring plan consists of clinical and behavioural observations according to the species and individual characteristics. Monitoring programs should include both short-term (i.e., during or immediately after the session) and long-term assessments (for the entire duration of the AAI project and throughout the animal life) [44].

The aim of monitoring plan is to detect both negative (“stressful”) and positive effects resulting from the interaction with the handler and the participant [97, 98].

Monitoring strategies, based on behavioural observations, are easy to perform and with a good cost-effective balance; however, they have some limitations because their reliability is strictly dependent on the dog handler’s competencies that can influence observations and interpretations [99]. Indeed, the handler should identify the signs of stress, monitoring the frequency and duration. In dogs, the most common are yawning; looking elsewhere/turning the head away; lifting back leg up; crying/complaining; increase respiratory rate; nose-licking; excessive salivation; scratching; stereotyped behaviours; aggressive and avoidance behaviours; anomalies in major organic functions; increased or reduced arousal, motor activity or appetite [100]. However, stress signs have a high inter-individual variability and low specificity; therefore, the handler should be very familiar with his/her animal [99]. The early recognition of stress signals allows the quick handler’s intervention to recover the animal psychophysical homeostasis and well-being, avoiding conditions of overstress and distress. Moreover, during AAI sessions, dog motivation should be enhanced through positive emotions. Therefore, the monitoring plan also includes signs of well-being that should be recognised by the handlers. The relationship between the dog and the handler is a key element for both the emotional balance of the dog and dog positive social interactions with the patient/client [101], with the handlers acting as a safe haven and a secure base for the dog [102, 97]. Indeed, social and environmental settings of AAI might cause stress to the dog due to unpredictable, uncomfortable events, unknown and/or fear-producing stimuli. Even if available data do not allow for conclusions about the impact of dog assisted interventions on the dog welfare, due to high heterogeneity of monitoring techniques, participants’ characteristics and AAI programs, a deeper knowledge is expected to be developed by future research [100].

### Dog health monitoring and eligibility

The dog’s health status should be carefully assessed by the veterinarian with expertise in AAI, who is responsible for the health monitoring program and procedures for the dog handler, which are necessary to manage the risks associated with the interaction of the dog with the older person. Eligibility of the dog is certified after a careful clinical examination, which must be conducted to rule out specific diseases (e.g., osteoarticular, cardiac, renal and gastroenteric diseases, otitis, conjunctivitis, dermatitis or other skin diseases). Further diagnostic testing should be performed, if deemed appropriate. The dog’s paws should be carefully evaluated, and nails should eventually be trimmed. Oral and dental care should be assessed to check for tartar, plaque and inflammation. Finally, the animal should have a normal weight according to the Body Condition Score (BCS) results.

The handler is responsible for care of the dog’s skin and coat (regular grooming and dead fur removal), eyes, ears and oral hygiene. Intimate hygiene should be performed routinely using sanitizing wipes. Moulting dogs should preferably have no access to healthcare or social facilities.

The dog must receive a homebased or commercial diet including cooked food, while raw or undercooked food are not allowed. Therefore, the BARF (Biologically Appropriate Raw Food) diet is not appropriate for dogs involved in DAI.

Health monitoring programs include periodic veterinary assessments, according to the duration of the intervention and the age and remote anamnesis of the dog involved. Laboratory testing is also performed, based on individual’s risk factors and on the characteristics of the setting [99].

### Health hazard of dog assisted interventions for older persons

The main hazards due to dogs in DAI programs for older persons include falls, scratches and bites. Dogs with a tendency to use their mouth or forelimbs for seeking attention should be excluded from these projects, as well as dogs of medium-large size that tend to jump or pull on a leash or do not have a strong proprioception and body-size awareness, thus potentially causing falls [103].

In order to minimize these potential risks, dog education programs should be oriented to promote a safe interaction between the animal and the participants and dog behavioural profile should be carefully assessed, taking into consideration the characteristics of older persons participating in the intervention.

Biological risks should also be considered, as dogs involved in DAI may cause zoonotic diseases - particularly in older persons with immunity deficits - or act as carriers of multi-drug-resistant pathogens [104]. Infection control protocols directed to both animal handlers and healthcare workers involved in DAI are essential to minimize biological risks.

### Risk assessment

Risk assessment requires detailed information on participants’ health status – provided by healthcare professionals - and on the environment where the intervention will be developed. Characteristics and life habits of the animal/handler team should also be considered as well as any other contexts in which they operate [102]. Finally, information about the dog’s remote anamnesis must be collected, including previous diseases, recurrent pathologies, antibiotic/recurrent pharmacological therapies, adherence to infection control procedures and prophylaxis. Risk assessment can be performed using qualitative or semi-quantitative methods and it should be based on the accurate collection of information about participant’s health status and environment.

### Control of ecto- and endoparasite infestations

The control of tick and flea infestations in dogs is crucial to minimize the risk of human exposure to specific pathogens that cause zoonotic diseases, including Lyme disease, Rickettsiosis, Ehrlichiosis, Babesiosis, and Bartonellosis. Although pets do not transmit arthropod-borne diseases to people, they can bring zoonotic disease vectors into domestic environments [105].

The General Recommendation of the ESCCAP (European Scientific Counsel Companion Animal Parasites) guidelines recommend year-round treatments against fleas and ticks using ectoparasiticide products with repellent and/or insecticide-acaricide activity as the risk of infestation is constant and the exposure is difficult to avoid [106].

Even though flea infestations peak in summer and autumn, studies have shown that flea infestation can occur throughout the year, thus year-round flea control might be necessary [107]. Tick prophylaxis should cover the entire period during which ticks are active. Depending on the level of risk and local legislation, this may consist of regular checking of the pet for ticks and/or acaricidal treatment. Currently, there are several ectoparasiticide products available on the market with insecticidal and acaricidal activity that the Veterinarian can select based on the specific needs and intervention strategies. Topical products, like spot-on, collars, and sprays formulated with synthetic pyrethroids, which are distributed through the skin’s lipid film on the dog’s hair and skin surface, providing a repellent and insecticidal effect after contact with the parasite or after ingestion of the active substance during blood meals. The systemic products (oral tablets and spot-on) require the parasite to begin feeding on the host to manifest their effect [108]. In dogs treated with topical products (spot-on) it is advisable to avoid touching/petting the dog in the first 48 h after the application of the product [109]. This precaution is not necessary with orally systemic products administered for oral way, as they distribute through the sub-epidermal blood plexus avoiding any risk of contact with the active substance [108].

Protecting dogs from sandfly bites is crucial due to the proven role of sandflies as vectors of leishmaniasis. Canine leishmaniasis is endemic in southern Europe with prevalence rates of infection of up to 60% in exposed populations [110]. As dogs are the main reservoir of *Leishmania infantum*, routine diagnostic tests are recommended for all dogs involved in AAI. This testing should be tailored to the geographical area and the dog’s lifestyle [109]. According to ESCCAP and LeishVet guidelines, all dogs living in or travelling to endemic areas should be protected with slow-release pyrethroid-based products, in the form of impregnated collars, spot-on and spray formulation that have demonstrated insecticidal/repellent activity against sandflies, throughout the entire period of vector activity. The basic objective is to interrupt parasite transmission and thus control the disease. The phlebotomine season in endemic areas may vary from year to year and from region to region. In some areas it may be necessary to protect dogs all year round (e.g., in southern Spain, Italy, Portugal and Greece) [110]. The continuous and widespread use of repellent and insecticidal products not only protects the dog from the risk of infection but also provides a level of protection against leishmaniasis in humans by reducing the reservoir and the number of infected sandflies [111]. At present, the scientific community agrees that the “combined” use of pyrethroids alongside the vaccination of “healthy” dogs represents the most effective approach for controlling the infection and the disease.

To reduce the risk of transmitting zoonotic endoparasites to humans, it is crucial to establish a comprehensive monitoring and treatment plan for dogs involved in DAI [109]. Recent studies evaluating the presence of potential endoparasites in animals involved in DAI have highlighted the presence of zoonotic intestinal helminths and protozoa in 24.3% and 30.4% of the subjects, respectively. The most found parasites were identified as *Giardia duodenalis*, *Toxocara canis*, and Ancylostomidae [112, 113].

While direct contact with parasitic elements in the environment is the primary route of infestation for humans, studies have shown that the presence of embryonated ascarid eggs or Giardia cysts on a dog’s fur can also serve as a source of infestation for humans through oral-fecal transmission [114].

The ESCCAP guidelines recommend a monthly copromicroscopic examination and treatment based on the results, to determine the most appropriate anthelmintic and establish the frequency of administration. Current information suggests that annual or twice-yearly treatments do not have a significant impact on preventing patent infection within a population. Therefore, a treatment frequency of at least 4 times per year is a general recommendation [115].

*Dirofilaria repens* and *Dirofilaria immitis* seem associated with climate change and a spread from historically endemic countries in Southern to Central Europe was observed [116]. Both species are known to be zoonotic, even though *D. repens* causes most infection; *D. immitis* infections are less frequent in Europe, but some cases have been reported even recently [117].

Hence, prophylactic treatment is recommended for all subjects involved in AAI [109] in order to guarantee the dog’s health and reduce the risk of transmission to humans, who can become infected following the bite of an infected mosquito that has fed on a microfilaremic dog [118].

In the last 10 years, in addition to cases of infestation by *D. repens*, which appeared to be the only species capable of infesting humans, several cases of human dirofilariasis caused by *D. immitis* have been reported in Italy, Greece, and Spain, with a growing trend in countries of Central and Northeastern Europe [119].

Current guidelines on management of *D. immitis* infestation in dogs promoted by ESCCAP and ESDA suggest extending chemoprophylaxis with macrocyclic lactones up to 7–8 months or even year-round due to the emergence of new vector species, such as the tiger mosquito (*Stegomyia albopicta*), which may survive in temperate areas as adult during winter [110, 119].

### Vaccination prophylaxis

In the context of vaccination prophylaxis for infectious diseases, the veterinarian must ensure that the dog involved in DAI has the most appropriate vaccination protocol based on its age, lifestyle, and risk of exposure. Particular attention should be given to zoonotic infectious diseases.

The guidelines on vaccination protocols are provided by the WSAVA (World Small Animal Veterinary Association), which national associations of private veterinarians refer to, adapting them to different local needs and realities. According to the latest WSAVA guidelines [120], vaccines are divided into “*core vaccines*”, “*non - core vaccines*” and “*not recommended*”. “*Core vaccines* “ are those that all dogs should receive, after considering their lifestyle and the geographical areas in which they live or to which they travel, and in all parts of the world are those that protect against *Canine Distemper virus* (CDV), *Canine Adenovirus type 1* (CAV) and *Canine Parvovirus type 2* (CPV) and, in Italy, vaccines to protect against *Canine Leptospirosis* [121]. Leptospirosis in dogs is a life-threatening, zoonotic disease affecting most mammals including humans, that is widely distributed around the world. In countries or regions where *Canine Leptospirosis* is endemic, where implicated serogroups are known and where suitable vaccines are available, vaccination of all dogs against leptospirosis is highly recommended and the vaccines should be considered *core* in those places. Globally, vaccines for leptospirosis currently on the market are predominantly bivalent (protecting dogs against the *Canicola* and *Icterohaemorrhagiae* serogroups), trivalent (providing additional protection against the *Grippotyphosa* serogroup, no longer available in Italy), or tetravalent (additional protection against the *Grippotyphosa* and *Australis* serogroups) [121]. Since cross-protection has not been demonstrated among serovars belonging to different serogroups, it is strongly recommended to use L4 vaccines (containing serovars belonging to the 4 most common serogroups) and discontinue the use of L2 vaccines [122]. According to the ECDC (European Centre for Disease Prevention and Control), the estimated prevalence of human cases of leptospirosis in Europe is 0.20/100.000 inhabitants per year, likely underestimated with seasonal peaks in late summer and early autumn [123, 124]. Therefore, vaccination of dogs should always be regarded as a fundamental measure for risk management and is essential in DAI to protect both the animals and the users involved.

*“Non-core vaccines”* as those that should be highly recommended in animals whose geographical location and/or lifestyle (e.g. indoor-outdoor access, multi-pet household) places them at risk of contracting particular infections not designated as “core”, such as the vaccine against the complex of infectious respiratory diseases (*Canine Infectious Respiratory Diseases Complex*, CIRDC) and leishmaniasis in Italy [121].

In the case of the rabies vaccine, since Italy has been declared free from rabies since February 14, 2013, vaccination is not mandatory. However, possible movements of animals within the country or abroad should be taken into consideration. Therefore, the potential vaccination of dogs should occur if the lifestyle of the animal and its handler could suggest an increased epidemiological risk, and in any case, if the animal travels beyond national borders [109].

### Further microbiological controls

Few studies investigated the hazard due to dogs involved in DAI transferring both zoonotic pathogens and nosocomial pathogens between individuals and the environment [125]. Preliminary data suggest that dogs are both a potential source of and a vehicle for the transfer of microorganisms to patients in institutionalised contexts, but not necessarily the only source [126].

Therefore, even if hygienic procedures during interactions, accurate grooming and cleaning of dogs before and after the interventions and accurate dog daily life management are the pillars to guarantee the safety of older people involved in dog-assisted interventions, additional microbiological control to monitor bacterial zoonotic agents and fungi that dogs can spread should be considered. *Salmonella spp.*, *Campylobacter jejuni* and *Campylobacter coli*, *Clostridium difficile* are suggested to be monitored in dog faeces samples to avoid possible outbreaks especially in preparation for interactions with frail or institutionalized older individuals [127]. *Pasteurella multocida* is a quite common agent in the oropharyngeal cavity of dogs [128]. In humans, it is associated with soft tissue infections or oral/respiratory tract infections mainly in older patients with chronic diseases [129]. Currently, antimicrobial-resistant ESKAPE (*Enterococcus faecium*,* Staphylococcus aureus*,* Klebsiella pneumoniae*,* Acinetobacter baumannii*,* Pseudomonas aeruginosa*, and *Enterobacter species*) pathogens represent a global threat to human health [130]. The risk of dog-human transmission and vice versa concerns all these bacteria and needs to be considered in surveillance plans according to the specific characteristics of the setting and to the environmental monitoring set up in the healthcare facility [131].

## Conclusions

The growing awareness about beneficial effects on human well-being of human-animal relationship has progressively increased the attention about this topic both in younger and older people. Some evidence is available regarding about the impact of human-animal relationship, but further studies are needed especially among older adults. However, encouraging results were provided by some preliminary studies investigating pet companionship and AAI in old age. Available data on pet companionship suggest that living with pets might have beneficial effects on cardiovascular risk, pain perception, emotional and mental wellbeing. Available evidence about AAI (services provided by professionals with the involvement of domestic animals) highlighted a potential on older individuals’ psycho-physical well-being in different settings of care. Expert’s support is required to ensure and maximize these effects and avoid risks for both older adults’ and animals’ health and well-being. Pet companionship may be challenging for some older adults. Counselling is suggested to determine the most suitable species for each individual and learn how to proper take care of it. AAI should be provided by specialised and trained professionals, respect some technical requirements and include an accurate risk assessment, in order to guarantee their quality and safety.

## Data Availability

Not applicable.
